# Analysis of the Doctor of Public Health (DrPH) training and identity needs in the United States: a qualitative study

**DOI:** 10.1186/s12913-023-10227-x

**Published:** 2023-11-07

**Authors:** Chulwoo Park, Cindy Delgado

**Affiliations:** 1 Department of Public Health and Recreation, San José State University, San Jose, CA USA 1 Washington Sq, 95192; 2 Cindy Delgado, Claremont Graduate University, Claremont, CA USA

**Keywords:** Doctor of Public Health (DrPH), DrPH graduate, Qualitative study, Focus group discussion, In-depth interview

## Abstract

**Background:**

The Doctor of Public Health (DrPH) is the highest attainable degree in the field of public health, specifically designed to prepare professionals to address complex public health challenges in practical settings. This study was designed to explore the importance of achieving a shared and uniform understanding of DrPH education, assess the optimal direction for DrPH training, and investigate the specific curriculum requirements by gathering insights from current DrPH students and alumni in the United States.

**Methods:**

A total of 13 focus group discussions and two in-depth interviews (total participants: 50) were conducted through Zoom to see how DrPH students and alumni assessed their DrPH educational programs.

**Results:**

Three overarching findings emerged from the analysis of focus group discussions and in-depth interviews. First, participants expressed a preference against a national DrPH board examination, but advocated for a standardized common core curriculum that extends across the entire nation. Second, the ideal direction for DrPH training was perceived to involve a practice-based approach, emphasizing the importance of multi-, inter-, and trans-disciplinary instruction delivered by faculty with practical experience. Last, there was a demand for a DrPH-specific unique curriculum encompassing areas such as mixed method analysis, leadership and management, applied communication, crisis and change management, proficiency in addressing contemporary topics, and tailored applied and integrative learning requirements specific to the DrPH program.

**Conclusions:**

We explored a range of DrPH training and identity needs among 50 participants, comprised of students and alumni who directly benefit from DrPH education. By considering these inputs, individuals from institutions that offer the DrPH degree can further enhance the quality of public health practice training and make significant contributions to the overall advancement of the field of public health.

**Supplementary Information:**

The online version contains supplementary material available at 10.1186/s12913-023-10227-x.

## Introduction

### What is DrPH?

For over a century, the Doctor of Public Health (DrPH) has been the terminal degree for the field of public health [1–3] that embraces professionals from various backgrounds. The DrPH was conceived to create public health leadership pathways and train competent leaders capable of leading public health by equipping them with the necessary skills [2–8]. However, one of the key challenges this degree faces is the lack of shared understanding of its existence, career outcomes, and intended purpose in public health education and practice [2]. Historically, the DrPH degree was conceived to train medical doctors, veterinarians, and dentists to become public health practitioners and leaders beyond their clinical training [8]. The DrPH was originally conceptualized based on the Doctor of Medicine (MD) training model to require a wide range of courses and applied, hands-on training similar to residency, but distinct from research-based degrees such as a PhD [8]. Over time, the DrPH has become a distinct professional interdisciplinary degree that prepares individuals for positions in leadership, policy, advocacy, community, and much more to work in transdisciplinary (researchers work together and integrate methods) and multi-disciplinary settings (researchers work independently to address a common problem) [9]. In this way, the DrPH eventually encourages interprofessional collaboration in education, research, and practice.

### Challenges of DrPH

A pervasive historical challenge the DrPH faces is inconsistency in degree requirements [1–3, 8, 10–13]. For instance, several DrPH programs continue to rely on research-focused models of training, which do not show clear differences from a PhD. Lack of distinction could create challenges for institutions to hire experienced senior-level practitioners with applied research who would teach and mentor DrPH students [4, 12]. Other challenges are workforce gaps in training and practice [12, 14], and limited capability to train leaders who can address the complexity of the social determinants of health [7]. To solve inconsistencies and curriculum issues with public health degrees, the Council on Education for Public Health (CEPH) created competency criteria for curriculum to help ensure public health programs were meeting the standards. Because CEPH criteria provided flexible guidelines, each program can interpret and implement CEPH criteria differently. As a result, all CEPH-accredited DrPH programs can meet the requirements but have different coursework and design. Although CEPH accreditation was intended to make a step forward for standardizing DrPH curricula, it appears that gaps and inconsistencies continue to exist due to institution-based interpretations, needs, and program structure. Despite standardization efforts, DrPH programs have continued to show similarities to the PhD degree, which contributed to the lack of clear distinction for the DrPH degree and the skills offered to students. Still, little is known about how professional doctoral education in public health should successfully train graduates to solve unexpected complex public health issues on the ground.

While previous research in this field has primarily relied on the professional opinions of DrPH program directors and individuals in leadership positions [2, 3], there has been a noticeable lack of peer-reviewed research studies addressing the needs of DrPH education recipients through a qualitative study. To address this gap, our study was designed to prioritize the perspectives of invested stakeholders: DrPH students and alumni who directly benefit from the DrPH degree. We aimed to understand and identify training and identity needs related to DrPH programs in the United States through the perspectives of DrPH students and alumni.

## Methods

### Study design

Focus group discussions and in-depth interviews were conducted online for each participant, via Zoom (Video Communications, San Jose, CA) from January 4 to 31, 2021. The inclusion criteria for participation was limited to current DrPH student or DrPH alumni in the United States, those who are primary receivers of the DrPH training. Participants from any of the DrPH programs were eligible to join, regardless of the CEPH-accredited status of their DrPH programs. DrPH graduates come with vast experience in public health, leadership, and management when entering DrPH programs. Thus, their diverse perspectives were important for us to understand the workforce needs, gaps, and future directions for the degree. Participant recruitment was carried out through a convenience sample of organizations, including the Student Assembly at the American Public Health Association (APHA), the DrPH Coalition (now known as the National Association for Doctors of Public Health, NADrPH)—a non-profit organization for DrPH students and alumni [15]—and DrPH directors in the United States. We contacted them via email and invited them to advertise and disseminate the online survey to potential participants through e-mail and social media (e.g., Twitter, LinkedIn) in November–December 2020. The research study was approved and received an exemption from the San José University’s Institutional Review Board.

### Study population

The online survey that we sent included a schedule of the focus group discussions, and potential participants selected their available time slots. A total of 95 potential participants showed interests joining our study, all of whom were invited. Among them, 50 participants took part in either focus group discussions or in-depth interviews. We conducted a total of 13 focus group discussions and 2 in-depth interviews. We grouped participants into two groups, Student Groups (SG, 8 groups with 29 participants) and Alumni Groups (AG, 5 groups with 19 participants) because we wanted to provide a comfortable environment for participants and assist peer group dynamics. Unexpectedly, the other two intended focus group discussions (one for SG, with five people invited, and one for AG, with six people invited) ended up being converted into in-depth interviews. This occurred because only one participant eventually joined each of the scheduled discussions.

### Data collection and qualitative analysis

At the beginning of the focus group discussions and the in-depth interviews, participants were asked to fill in the online survey, answering demographics questions and the following two binary questions: “Should there be a board certification of DrPH?” and “Should there be a standard national curriculum for the DrPH degree?” We designed focus group discussions to last a maximum of 90 min and tried not to exceed this expected duration to show the courtesy of promised time to participants. The structured interview guide was created so all interviewers were consistent, sharing the same script, prompt, and questions across all focus group discussions. Among the entire questionnaire, this study focused on the DrPH training, DrPH curriculum, and CEPH requirements. The list of CEPH competencies was displayed through “Share Screen” function on Zoom to help participants answer two relevant questions about alignment with CEPH standardization. We developed the questionnaire based on the topic areas and their key questions for this study (See Supplementary Table). Almost all the focus group discussions and in-depth interviews (14/15) lasted at least 60 min, ranging from 35 to 97 min, with an average of 78 min. All focus group discussions and in-depth interviews were visually recorded with the audio in the first author’s Zoom account. We did verbatim transcription for each of the focus group discussions and in-depth interviews to capture the exact quotations and avoid returning transcripts to participants for comment or correction.

To manage and analyze the data, NVivo was used (QSR International, Melbourne, Australia). Each of those verbatim transcriptions was stored in the individual Microsoft Word document (docx), and those documents (total: 15) were imported to NVivo. We approached both deductive and inductive reasoning while analyzing the transcripts. We first created themes, categories, and codes based on the existing questionnaire in NVivo for deductive reasoning, and then additionally worked on themes and coding that were newly discovered through inductive reasoning. Table 1 shows qualitative data analysis within each of the themes.

**Table 1:** Summary of coding analysis

Themes	Categories
Why pursue DrPH?	◦ Unique skills obtained from a DrPH ◦ Customization and flexibility
The necessity of nationwide DrPH common core	◦ Standardization for common core curriculum ◦ Standardization for entire curriculum ◦ National DrPH board examination
Ideal DrPH training direction	◦ Practice-based training ◦ Multidisciplinary, interdisciplinary, and transdisciplinary training ◦ Community-based partnerships ◦ Flexibility and customization ◦ Practitioner faculty
DrPH-specific unique curriculum needs	◦ Mixed methods analysis ◦ Leadership and management skills ◦ Applied communication skills ◦ Crisis and change management ◦ Cross-cutting contemporary topical training ◦ Integrative learning experience
DrPH identity needs	◦ Advocacy for better understanding of DrPH ◦ DrPH branding and marketing ◦ Strong connection between DrPH and public health system

## Results

### Demographic information from the online survey

Table 2 presents the demographic information of attended participants in focus group discussions and in-depth interviews, collected from the online survey. In addition, 80% (40/50) of the participants answered “Yes” to the question, “Should there be a standard national curriculum for the DrPH degree?” However, 66% (33/50) of the participants answered “No” to the question, “Should there be a board certification for DrPH?”.

**Table 2:** Demographic information of participants (*N* = 50)

Characteristic	n	(%)	Characteristic	n	(%)
**Age**			**Place of living**		
26–30	8	(16)	West	7	(14)
31–35	11	(22)	Midwest	3	(6)
36–40	10	(20)	South	14	(28)
41–45	6	(12)	Northeast	24	(48)
46–50	6	(12)	Outside the U.S	2	(4)
51–55	4	(8)	**Race**		
56–60	5	(10)	American Indian/Alaskan Native	2	(4)
**Gender**			Asian or Pacific Islander	5	(10)
Male	10	(20)	Black	19	(38)
Female	40	(80)	White	20	(40)
Intersex	0	(0)	Other	3	(6)
Other	0	(0)	Prefer not to say	1	(1)

### Why pursue DrPH?

Participants were asked to answer how they decided to choose a DrPH degree over a PhD degree. Their answers were classified into the following two categories: a) unique skills obtained from a DrPH degree and b) flexibility and customization. Among the reasons for selecting a DrPH degree, participants mentioned hearing about the program by being recommended to it by their mentors and/or colleagues (Deans of the school of public health, department chairs, academic advisors, colleagues, and other recommended or advised participants), which significantly helped them to make a final decision to pursue a DrPH degree:

I applied to a PhD program in health policy administration, and . . . I was 36 or 37 when I applied. And the Chair of the department called [me] and said, “I think you’re one of our top candidates. . . . Why don’t you call the chair of the DrPH program and talk with them and see what you want to do?” . . . So I talked to the DrPH program [on] that very day . . . they said, “You’d probably fit better with a DrPH because all of them are kind of mid-career [with] several years of experience.” So they rejected me from the health policy and administration PhD so that my application can route to the DrPH, and then they had to accept me into the pool. (AG 5)

#### Unique skills obtained from a DrPH

There were varying reasons for selecting a DrPH over a PhD. The primary reason was to learn applied public health coursework and advance their leadership skills. Participants described selecting a DrPH for its flexibility and a wider spectrum of applied topic selection (e.g., the incorporation of transdisciplinary approaches) and other fields (e.g., psychology, emergency management, and change management). Additionally, they expressed their interests in advancing their careers and knowledge through applied research skills and analysis. For many participants, the DrPH degree was expected to provide opportunities to advance their current leadership skills.

#### Customization and flexibility

While a PhD requires diving into a narrow area to become an expert on a particular subject, a DrPH allows a broader spectrum of topic customization for a transdisciplinary approach. Participants who wanted to pursue a wider scope of public health decided to earn a DrPH degree:

A PhD is really, you get a topic, you narrow that topic, and you do a deep dive as an expert into a very narrow area. . . . And DrPH, we have more of a breath, we have a wider spectrum of topics. . . . I just can’t imagine having a narrow topic and spending that much time looking at one little thing. I don’t even think I could bring my mind in enough to do that. So I knew that the DrPH was for me. And I think it’s one of the best decisions I ever made. (AG 2)

Many DrPH programs had no fixed time duration for completion of the study. Just as a PhD degree, the years to complete a DrPH degree depends on when students are able to finish their dissertation. However, some DrPH programs provided an executive DrPH leadership program that requires a shorter period than that in PhD programs. Some participants mentioned that a DrPH degree was a good fit for them because it only takes 3–4 years to complete, or it was expected to be completed earlier than a PhD program. Allowing them to continue working full-time in Senior Leadership positions:

I didn’t want to spend seven years doing a doctoral program [(PhD program)] when I’m almost 50. I’m in an accelerated [DrPH] program. I’m done with my coursework in two years, and then, however long it takes me to do my dissertation research and write up, which is totally on me but realistically, I can be done in three and a half years. (SG 6)

### The necessity of nationwide DrPH common core

#### Standardization for common core curriculum

We asked to what extent participants would think DrPH curriculums across the schools or programs in the United States should be standardized, just as a PhD program, from coursework to dissertation defense. Most participants expressed a desire for the standardization of at least the DrPH common core curriculum, which includes biostatistics, epidemiology, social and behavioral sciences, health services administration, and environmental health sciences. However, they did not want the entire program to be standardized nationwide. Although each DrPH program has its own culture and different administration, there should be shared commonalities to establish the conformed DrPH identity. After learning standardized core in DrPH courses, participants wanted to tailor their concentration or specialization to build up a specific skill set:

I think there should be some core elements that are standardized across programs and then have the opportunity to tailor based on concentration or any institution specific. I think it would help if there was a core part of [the] curriculum that remain[s] consistent between institutions. (SG 8)

#### Standardization for the entire curriculum

Unlike the demand for standardization of the DrPH common core curriculum, participants did not reach a consensus on the necessity of standardizing the entire DrPH curriculum nationwide. Some participants wanted to see entire curriculum standardization across the country because it is inequitable that each DrPH program has a different time duration for graduation due to different interpretations of the DrPH integrative learning experience:

If you look at [specific DrPH programs], you need a lot more experience, but then you look at the curriculum, and their program only [needs] three years. They do a culminating experience like [a] type of Capstone Project. [However,] a lot of us [from different DrPH programs] are either doing a dissertation or have to do a dissertation. So it will definitely be a five [or] four-plus year type of program. . . . It needs to be consistent across the [country] because it’s definitely not fair that folks start doing the same degree, but it’s taking longer because the requirements are different. (SG 5)

On the other hand, others mentioned that there were already many prerequisites and requirements to become qualified to be a DrPH applicant, such as an MPH degree and work experience. Thus, they wanted the flexibility of pursuing their specialization:

In the program that I went into, you had to have your MPH to get into the DrPH program. And I feel like there is a lot of standardization in the MPH programs which gave you the foundations of Public Health. So I don’t see that as needing to be continued into the DrPH. You can specialize a path, like a PhD . . . There needs to be flexibility still within the DrPH. (AG 1)

#### National DrPH board examination

During focus group discussions and in-depth interviews, we asked participants’ opinions about having a national DrPH board examination in greater detail. Most participants disagreed or had mixed feelings about establishing a board examination for a DrPH degree. For instance, some questioned the practical value of having a board examination for the degree. Financial barriers were mentioned as a concern, especially when its use might only serve to maintain their status and membership in the group. In addition, others described it would be challenging for a certification to cover the different topics and variabilities that exist within DrPH programs and public health in general:

I personally just don’t believe that a certification exam and certification is necessary. I think it would be very challenging to do. Not just because of the variability of programs, but also the variability of public health in general. It is very difficult to learn all of everything. Do you have to learn everything about injury health, environmental health, occupational health, community health, infectious disease, [and] epidemiology? (SG 8)

### Ideal DrPH training direction

#### Practice-based training

The majority of participants expressed interest in coursework with a focus on the practice and real-world application outside of academia. Participants expressed that the CEPH competencies seemed distant from systematic practice-based approaches combined with theorical focus. For participants, the lack of integration was creating a gap in their training:

It’s boots on the ground. . . . What drew me to practice is because you can have those true academics, and researchers, and the people in the labs under the microscopes. And we need all of that, but I think you have to couple with what’s happening out there. . . . I’m going back to my CBPR [(Community-Based Participatory Research)] principles, but that’s often the voices that we need to hear that aren’t always heard. (AG 6)

#### Multidisciplinary, interdisciplinary, and transdisciplinary training

Public health practice needs to act as multi- and inter-disciplinarity, such as the intersection between policy and healthcare delivery. By extension, the DrPH program needs to pursue a transdisciplinary approach. Rather than focusing on a narrow topic, a DrPH degree should allow participants to choose a wider spectrum of skills needed in public health. In addition, depending on students’ interests, the DrPH curriculum should be customized:

I describe as PhD is really, you get a topic, you narrow that topic, you do a deep dive as an expert into a very narrow area. And DrPH, we have more of a breath, we have a wider spectrum of topics. . . . I just can’t imagine having a narrow topic and spending that much time looking at one little thing. . . . So I knew that the DrPH was for me. And I think it’s one of the best decisions I ever made. (AG 2)

#### Community-based partnerships

Another training direction that participants wanted to see was collaboration and partnership with communities as a common and vital component of the response to community health practice. As a result, some participants described the importance of community-based participatory research (CBPR) and community involvement, such as SG 6’s thought, “Community health needs assessment [is needed to] work with stakeholders in the community, about what they need versus what can we provide.” In addition, participants expressed their eagerness to understand how to establish and collaborate with communities and multiple-level partnerships:

I need to know how to navigate multi-sector partnerships. And how do you bring people to the table? How do you navigate conflicting interests or tensions? How do you bring together funding streams? How do you navigate politics? How do you advocate for policy change? Who were the people, that you should be going to advocate for those changes? What are the most effective pathways? These are all kinds of things that we should be taught in a DrPH curriculum. (AG 2)

#### Flexibility and customization

Many participants had full-time jobs outside of their DrPH program. Some of them wanted to have more online classes to accommodate their needs of working while studying. People in the military or the independent federal government agency wanted to be more flexible to customize the coursework remotely while working full-time at different places:

For me, it would have been nice to have other online courses . . . because [the] Department of Defense at the federal level actually [has] quite a few people [who] work in public health. And they move around, so they don’t want to stop their degree program. We [also] had people that work for USAID and were in Africa. Some of these people were taking [a] class at two in the morning, synchronous sessions online, but they were able to still get their education, still do really good public health work in the organizations. (AG 5)

#### Practitioner faculty

Many DrPH courses have been taught by faculty with a PhD degree. However, the majority of participants expressed the view that faculty with a PhD degree may not always be equipped to focus on public health practice, necessitating a holistic new approach in teaching DrPH courses. Over half of DrPH programs in the United States mandate prior work experience in the field of public health as a prerequisite, with some specifying a requirement of several years of postgraduate or full-time work experience [2]. Some DrPH programs are designed for mid-career public health practitioners seeking to enhance their knowledge, practice, research, and application [16, 17]. Participants desired to see more faculty with a strong public health practice perspective who can teach and guide them as a mentor or an academic advisor:

I didn’t know anybody at (school name), and this small number of core faculty for the DrPH weren’t able to help me because they didn’t have enough expertise. . . . We ought to have somebody in our associate program that can help us with data problems, right? (AG 5)

Some alumni wished to know how to support faculty who need more capacity to design coherent coursework for their DrPH program, such as reflecting the expectations of both research and practice:

How can we help the faculty do their jobs better? Nobody wants to say, “I took six courses here, and it was all irrelevant, or it was outdated, or whatever.” I’m sure there are other people like me that are really interested in the intersection between research and practice. How can we help write up the case studies for somebody’s course? (AG 5)

### DrPH-specific unique curriculum needs

#### Mixed methods analysis

While some participants desired a curriculum with a stronger focus on practical experience, others expressed a need for greater exposure to mixed-methods analysis due to the absence of qualitative or mixed-methods courses. AG 1 stated, “I think there should be a greater emphasis on preparing DrPH students as a mixed-method, or I find... lack [of] the qualitative portion. If I wanted to take a qualitative course, I would have to take it in a different school.” SG 6 also noted, “If I’m a practitioner, I want to apply different types of methods.... I want to take mixed methods classes, which we don’t offer currently at (school name).” Additionally, participants expressed a desire to learn how to translate complex methods into the common language that the public can understand.

#### Leadership and management skills

Participants expected that the DrPH program should provide essential leadership skills to advance their executive positions in their workplace. Participants hoped to see more emphasis on leadership and management skills in their DrPH programs. They felt that developing those skills would differentiate them from all other professional terminal degrees. AG 3 mentioned, “The biggest difference [between the DrPH degree and other professional terminal degrees] is that MDs like doctors, physicians, and PhDs aren’t necessarily taught how to work with people, how to lead people, how to manage a budget, how to manage a process, [and] how to manage a project.”

#### Applied communication skills

Some participants highlighted the importance of developing the skill of writing, public speaking, and health communication, which would help them to approach various audiences for bridging between academia and the public. For example, AG 1 mentioned, “Health communication is critical.... If you’re going to be [in] a leadership position, I think that should be a forefront to your degree, the writing and health communication.”

#### Crisis and change management

Crisis and change management was identified as one of the crucial skills that participants wanted to acquire during their DrPH training. They believed that public health leaders should be adaptable in the face of sudden adverse events and should apply the skills they have learned when responding to unexpected consequences. As a result, participants suggested that crisis and change management coursework should be integrated into the DrPH program. AG 2 mentioned, “We make plans in just about every field or sector that we work in, but those plans don’t always work. Sometimes you have to modify and adjust those plans. I think as a public health leader, that’s a skill that you certainly must have, like crisis management.”

#### Cross-cutting contemporary topical training

Participants wanted to learn more about particular topics related to technical, practical aspects of public health, such as a) quality improvement, b) artificial intelligence and machine learning, c) lobbying the budget, d) health equity and health disparities, e) implementation science, f) scaling up intervention, g) climate change, h) monitoring and surveillance, i) public health law, and j) public health ethics. It is important for DrPH graduates to apply those contemporary topics in practice. For example, SG 4 observed, “We have cultural values, but I think more anti-racist frameworks need to be integrated in[to] the DrPH, especially if we’re leading organizations and managing people with different backgrounds. And we need to be able to address any of the personally mediated racism or colorism.”

#### Integrative learning experience (Dissertation)

Many participants pointed out that their DrPH programs did not differentiate the DrPH integrative learning experience (e.g., dissertation) from the PhD dissertation. They expressed that their dissertation requirements and formats reflected the lack of difference between a PhD and a DrPH. SG 5 mentioned, “I’m in the dissertation phase and actually... if you put a PhD dissertation and the DrPH dissertation, you won’t see any major difference... I want to do a practice-oriented dissertation. It should be structured separate[ly] from a PhD dissertation.” Participants also urged to develop DrPH’s unique common core curriculum, assessment of core competencies, residency and practicum, and community activity through an organizational shift and structured involvement:

[It] makes me think of going back to the residency and practicum ideas that is a structured way to involve students and working professionals who are interested and a course of helping a project or a study or community activity. I think that’s a good way of building it [be]cause it really gets in your bones once you’re actually do[ing] that work. (AG 3)

### DrPH identity needs

#### Advocacy for better understanding of DrPH

Participants mentioned that the public usually is not aware of the definition of a DrPH, which made them feel a sense of responsibility to explain what a DrPH degree is to the public. Alumni highlighted that advocacy would help establish the DrPH identity. Advocacy would help position a DrPH degree as a pathway to be in the leadership position, as a recognized public health terminal degree education, and as a guidance to pursue an interdisciplinary approach in solving public health issues:

Advocacy, especially at APHA [(American Public Health Association)]. We can advocate because they’re a big powerhouse, [so] once they make the issue statement, a lot of people kind of fall in line. . . . We’re just as competent as a PhD person. Whenever there’s a call for fellowship, there should be DrPH there as well. (AG 1)

It was also important for participants to advocate for themselves, speaking up about the DrPH degree at their workplace or institution, in order to help co-workers, individuals around them, and the public understand the unique role of DrPH. AG 2 noted, “Start having this conversation within your institution, whether it’s in your staff meetings. Don’t be the person just sitting at the table. Be more vocal, be heard.” After receiving their degree, they felt comfortable and confident to share and explain evolving COVID-19 information to assist the community in making informed decisions:

I have people coming to me with questions and now I’m able to utilize the knowledge that I learned in school and through my career experience and help people understand what public health is, how they can understand what’s happening with this pandemic, the numbers [(COVID-19 cases, mortality/morbidity rates), [and] the vaccine. That makes them feel comfortable coming to somebody who is giving [wrong] facts and correct information so that they can make better choices, whether it’s individually or within the community or the overall population. (AG 6)

#### DrPH branding and marketing

AG 6 highlighted the importance of establishing branding of a DrPH using metaphor, “If we were all to identify ourselves with something that you wear, or like a color. Whenever we get together, that will be our symbol. We believe in this, so we are DrPH.” Many alumni were willing to share their stories and experiences to the public regarding how they became interested in a DrPH to help creating a stronger identity of a DrPH degree. AG 6 mentioned, “I share my story now of the process that I went through to get it. As you’re asking these questions, why I got it and what I want to do with it, I think that’s the best way at this point to continue to create a stronger identity for this particular program.” Some alumni highlighted the importance of marketing to establish a DrPH’s identity. Each DrPH program can create its own DrPH club or organization just like a fraternity, then establish a network of connecting through school fairs or any other type of gatherings. This would also draw attention to people who would get to know a DrPH and pursue a DrPH degree:

I was more thinking along the lines of how our fraternity works, so you have your national headquarters and then you have your schools. For example, each school has a DrPH program [and] they can have their own little mini club, so to speak. They network with each other, and maybe they can go to school fairs and have little booths where they have all the information about the DrPH. (AG 6)

#### Strong connection between DrPH and public health system

Contribution to building public health infrastructure and systems was also frequently mentioned among participants. For example, AG 4 mentioned, “We are expected to go and actually be part of the public health system. From that applied point of view, I think it’s really important right now.” A participant from student group highlighted that DrPH graduates should be the one who fill the needs of public health workforce and engage in system-level conversations:

[At the] DrPH Coalition meeting at APHA 2 years ago, someone shared that only 13% of health administration leadership positions are held by people with public health degrees. I think that is something that needs to be filled by DrPHs and just being included on these big National Task Forces, thought leadership, especially about ending structuralisms, and really being that voice that can translate the research into practice. So I see DrPH people [should be] at the table of these big system-level conversations. (SG 1)

## Discussion

The DrPH is the professional doctoral degree in public health, aimed at nurturing transformative academic and practice leaders with expertise in evidence-based public health practice and research [18–20]. According to Park and Coles (2022), in light of the exacerbated workforce shortages caused by the COVID-19 pandemic, there was a pressing need to enhance recruitment efforts for the public health workforce, particularly in leadership roles, with DrPH graduates being considered potential contributors to addressing this gap [5]. Among the reasons for selecting a DrPH, it was clear that participants’ selection was grounded in their desire for advancing their current leadership skills and a career in public health leadership. For some participants, this meant learning applied research methodologies and applied data analysis that could answer social challenges in their communities and produce outputs to informed decisions around policy, leadership, and strategic programmatic thought leadership. Others focused on learning the art of disseminating information and strategic communication to effectively translate evidence into action and lead public health advocacy efforts. For the remaining participants, the DrPH was an opportunity to combine their current subject matter expertise such as psychology, emergency management, medicine, nutrition, and behavioral health with DrPH specific skill sets in leadership and management to competently engage in a transdisciplinary practice that would allow them to expand their social impact beyond a siloed niche. Participants found this synergy to be a unique strength of a DrPH credential that allowed them to serve as chief public health strategists.

Our findings contributed to discussing standardization efforts needed for DrPH programs [6, 12] and revisiting the challenges they have faced when meeting the training needs of public health practitioners in the twenty-first century [21]. For example, the DrPH education can cover both methodology and its application on the ground to address timely special topics, such as the use of qualitative and quantitative research skills for mental health in war and conflict [22]. Another example of this need is that the perceived usefulness of formal learning was positively associated with employee’s three employability competences, occupational expertise, anticipation and optimization, and personal flexibility [23]. Through the terminal degree in public health, DrPH, students can gain new knowledge and skills to enhance employability competences.

Among participants in this study, the DrPH gave them the recognition they needed as an established public health leader. The program gave them the unique, transferable, and diversified skills needed such as communications, mixed methods, policy and program development, evaluation, and implementation to advance in their careers and opened new frontiers in a variety of fields. Several participants also expressed interest and professional experience in academia as a non-traditional approach. This highlights the need for structural changes in academia and a shift towards creating opportunities for practitioners and adjunct professors to take on faculty roles in training the next generation of public health leaders and professionals. These findings also underscore the academic orthodoxy of rewarding researchers and the need for a more balanced approach to recruiting and valuing practitioners.

Participants suggested that DrPH’s distinct identity should be systematically established and shared through branding and marketing strategies. To establish strong identity of a DrPH degree, various suggestions were made including storytelling (e.g., sharing their leadership stories and journeys), creating symbols of recognition when attending events, supporting faculty who teach DrPH courses, marketing DrPH through a club or an organization, and establishing DrPH’s common core curriculum. Additionally, the DrPH degree could be closely integrated with or adapted to the structure, needs, and requirements of the broader public health system. This alignment could involve curricular and organizational changes that make the DrPH program better resonate with and serve the public health system’s goals and objectives.

Still, several challenges persist in establishing DrPH training, particularly regarding the extent of standardization within the DrPH curriculum. This discussion centered on whether the standardization should exclusively focus on the DrPH common core curriculum encompassing the traditional public health core knowledge areas that all DrPH students in the United States need to learn (i.e., biostatistics, epidemiology, social and behavioral sciences, health services administration, and environmental health sciences) [19] or if it should extend to cover the entire DrPH program, including the nature of the DrPH integrative learning experience. Three approaches can be considered to address this issue further. First, if educational institutions of public health aim to position the DrPH as an applied degree, they can consider the needs of practitioners in the field. It is crucial to explore ways to design a multi-disciplinary, interdisciplinary, and transdisciplinary curriculum that aligns with the collaborative nature of DrPH roles and the needs of change agents, and integrate the essential Certified Health Education Specialist (CHES) and Master Certified Health Education Specialist (MCHES) competencies. Second, since DrPH graduates are the direct beneficiaries of educational training, their perspectives can play a more significant role in curriculum design and program structure to adequately address their training needs in leadership, management, and cross-cutting topics. Finally, DrPH institutional models following research-focused training approaches can consider the need for flexible and online curriculum options to accommodate full-time working professionals pursuing a DrPH degree.

Furthermore, it is essential to delve into similar challenges faced by other health professions education programs, such as Doctor of Education (EdD), Doctor of Psychology (PsyD), Doctor of Social Work (DSW), and Doctor of Nursing Practice (DNP). Each of these programs encounters distinct but interrelated issues that bear similarities to those faced by DrPH education. For instance, EdD programs may face challenges related to curriculum design and alignment when transitioning into professional practice doctorate programs [24]. Similarly, PsyD programs need to strike a balance between providing extensive clinical training and fostering a robust theoretical understanding to adequately prepare psychologists for the diverse needs of patients [25, 26]. DSW programs have recently experienced a resurgence to bridge the research-practice gap by placing a primary emphasis on clinical practice and leadership within their curriculum. Unlike traditional doctoral programs, DSW programs do not require a qualifying examination, and graduation typically depends on the completion of a capstone project or portfolio instead of traditional dissertation [27, 28]. Challenges throughout the different stages of DNP programs include defining practice gaps and lacking training in protocol development during the design stage, struggling with project site access and mentorship during the implementation stage, and facing issues with project evaluation criteria and the use of quality improvement measurement tools during the evaluation stage [29]. Additionally, overarching challenges include difficulties in scholarly writing, faculty preparation, and project sustainability, all of which hinder the successful completion of DNP programs [29]. Exploring these commonalities is essential not only for comprehending the broader issues impacting health professions education but also for formulating comprehensive solutions that can enhance the effectiveness of doctorate training across multiple health-related disciplines.

### Limitations of the study

This study included a few limitations. First, two scheduled focus group discussions (one student group and one alumni group) ended up having only one participant. We expected that all participants who were invited to those focus group discussions would join Zoom later while the discussion was ongoing; however, they became an in-depth interview because no one else joined until the end of the discussion, even though we sent an email reminder to invite participants before focus group discussions. While these individuals shared their opinions and thoughts about the DrPH program in the United States, there was a possibility that their perspectives might be biased towards their specific DrPH program, making them less representative of the broader group, including all other DrPH graduates from those programs. This unexpected situation was related to the limitation of online focus group discussions and in-depth interviews because we relied on participants’ email addresses for communication and their acceptance response to the Zoom invitation without knowing whether they would participate or not.

Second, the opinions of participants in our study may not represent the entire DrPH community in the United States. Most participants were from the Northeast (48%), South (28%), and West (14%) regions of the United States, so they may not be representative of the whole DrPH experience. Future research can consider the voices of those less represented regions and incorporation of individuals who are in new programs.

Third, additional challenges come from the voluntary nature of those who self-selected to participate in the focus group discussions; thus, future research can consider probability sampling techniques.

Fourth, we did not ask a question about the MCHES designation and its significance regarding the responsibilities, competencies, and sub-competencies outlined within the Advanced 2 designation framework published by the National Commission for Health Education Credentialing (NCHEC) and the Society for Public Health Education (SOPHE) [30]. Future studies can investigate how the DrPH curriculum incorporates the eight areas of roles and responsibilities from MCHES, including assessment of needs and capacity, planning, implementation, evaluation and research, advocacy, communication, leadership and management, as well as ethics and professionalism [30].

Fifth, this study may not fully consider potential confounding factors affecting both individual student and/or alumnus perceptions and biases, as well as the complex challenges confronting health professions education across various disciplines today [31]. These intricate issues encompass the dynamic landscape of evolving healthcare systems, shifting societal demands, technological advancements, and health services [31], all of which can significantly shape not only the perspectives of each student and alumnus but also the overarching context in which DrPH education is situated. Thus, it is imperative to recognize that the findings in this study should be interpreted within the broader context of the ongoing complexities facing health professions education on a larger scale.

Finally, with regard to the preparation of a questionnaire in November–December 2020, we expected the COVID-19 pandemic would not last long, and we did not ask specific questions about COVID-19 impacts around the DrPH program. We could have asked participants to discuss the role and expectations of DrPH graduates for controlling COVID-19 as a way of increasing DrPH perception. Future research can consider analyzing the interest and needs of DrPH graduates to identify gaps and workforce requirements arising from stakeholders and employers, particularly during complex public health emergency. This analysis should encompass a broader range of situations, including but not limited to various types of disasters and global public health challenges and issues, such as disease outbreak or war on a global scale.

## Conclusions

A DrPH degree provides unique skills that a traditional PhD degree has not focused on, such as leadership and practice, transdisciplinarity, health management application, applied research, and policy implications. This needs assessment from the perspectives of DrPH invested stakeholders will continue to inform existing and future DrPH programs. The majority of participants expected that faculty with practical experience would serve as mentors or academic advisors for DrPH graduates. For DrPH training and curriculum needs, flexibility to tailor training should be ensured, while standardizing the core DrPH curriculum. However, it remains to be seen whether standardization of the entire DrPH curriculum until graduation is necessary. Students should learn cross-cutting issues and frameworks such as anti-racism, global health equity, and climate change as the core of DrPH training. Practice-based “boots on ground” training that emphasizes a diverse skill set is needed, such as change management, financial leadership, mixed-methods training, crisis management, and communication sciences. Multidisciplinary training can equip DrPH students to formulate innovative, trans- and inter-disciplinary policy and programmatic solutions. Leadership training should focus on self-reflection, ethics, philosophical groundings, and critical thinking. An integrative learning experience should build on connection across seemingly disjointed discipline-specific skills. The future contribution of DrPH graduates will bring recognition to the field of public health by creating a strong public health voice and workforce. It is expected that DrPH-offering institutions should reflect DrPH graduates’ needs to provide enhanced quality of public health training.

### Supplementary Information

**Additional file 1.**

## Data Availability

The datasets used and/or analyzed during the current study are available from the corresponding author on reasonable request.

## Abbreviations

DrPH: Doctor of Public Health
MD: Doctor of Medicine
CEPH: Council on Education for Public Health
SG: Student Groups
AG: Alumni Groups
CBPR: Community-Based Participatory Research
APHA: American Public Health Association
