# Supplementary material for: Analysis of the Doctor of Public Health (DrPH) training and identity needs in the United States: a qualitative study
Source: BMC Health Serv Res. 2023 Nov 7;23:1216. doi: 10.1186/s12913-023-10227-x (PMC10629150; doi:10.1186/s12913-023-10227-x)
Supplement: Supplementary file 1 — Additional file 1. [file 12913_2023_10227_MOESM1_ESM.docx]

**Supplementary Table.** The interview questionnaire

| **Prompts** | **Key questions** |
| --- | --- |
| Why pursue DrPH? | - How did you decide to choose a DrPH degree over a PhD degree? |
| DrPH Curriculum | - What is your reflection on your DrPH curriculum? - How should the DrPH curriculum be changed to make a clear distinction from a PhD degree? |
| Alignment with CEPH  Standardization | - Do you think CEPH competencies adequately reflect the skills needed for a trained public health workforce? - Do you think your DrPH program successfully has addressed those 20 CEPH competencies? **Note: We used the Zoom screen sharing to present this information to the participants.* |
| Standardization | - Do you think DrPH curriculums across the schools or programs in the United States should be standardized, just like a PhD program (e.g., from coursework to dissertation defense)? - Another example would be establishing a board certification exam. If yes/no, could you elaborate? |
| Identity | - How would you like to help establish the identity of the DrPH program? |
